# Attitudes of patients and family members towards deferred and waived consent in ECPR research, an ancillary study of the INCEPTION trial

**DOI:** 10.1016/j.resplu.2026.101239

**Published:** 2026-01-21

**Authors:** Stijn E.D.M. Eussen, Anina F. van de Koolwijk, Thijs S.R. Delnoij, Martje M. Suverein, Brigitte A.B. Essers, Renicus C. Hermanides, Luuk C. Otterspoor, Carlos V. Elzo Kraemer, Alexander P.J. Vlaar, Joris J. van der Heijden, Erik Scholten, Corstiaan A. den Uil, Dinis Dos Reis Mirada, Sakir Akin, Jesse de Metz, Iwan van der Horst, Bjorn Winkens, Jos G. Maessen, Roberto Lorusso, Marcel C.G. van de Poll, INCEPTION-investigators, Martje M. Suverein, Thijs S.R. Delnoij, Roberto Lorusso, George J. Brandon Bravo, Luuk Otterspoor, Carlos V. Elzo Kraemer, Alexander P.J. Vlaar, Joris J. van der Heijden, Erik Scholten, Corstiaan den Uil, Tim Jansen, Bas van den Bogaard, Marijn Kuijpers, Ka Yan Lam, José M. Montero Cabezas, Antoine H.G. Driessen, Saskia Z.H. Rittersma, Bram G. Heijnen, Dinis Dos Reis Miranda, Gabe Bleeker, Jesse de Metz, Renicus S. Hermanides, Jorge Lopez Matta, Susanne Eberl, Dirk W. Donker, Robert J. van Thiel, Sakir Akin, Oene van Meer, José Henriques, Karen C. Bokhoven, Henrik Endeman, Jeroen J.H. Bunge, Martine E. Bol, Bjorn Winkens, Brigitte Essers, Patrick W. Weerwind, Jos G. Maessen, Marcel C.G. van de Poll

**Affiliations:** t Department of Intensive Care, Maastricht University Medical Center, Maastricht, the Netherlands; u Department of Cardiothoracic Surgery, Maastricht University Medical Center, Maastricht, the Netherlands; v Department of Cardiothoracic Surgery, Isala Klinieken, Zwolle, the Netherlands; w Department of Intensive Care, Catharina Hospital, Eindhoven, the Netherlands; x Department of Intensive Care, Leiden University Medical Center, Leiden, the Netherlands; y Department of Intensive Care, Amsterdam University Medical Center location AMC, Amsterdam, the Netherlands; z Department of Intensive Care, University Medical Center Utrecht, Utrecht, the Netherlands; aa Department of Intensive Care, St. Antonius Hospital, Nieuwegein, the Netherlands; ab Department of Intensive Care, Erasmus Medical Center, Rotterdam, the Netherlands; ac Department of Intensive Care, HagaZiekenhuis, Den Hague, the Netherlands; ad Department of Intensive Care, OLVG, Amsterdam, the Netherlands; ae Department of Intensive Care, Isala Klinieken, Zwolle, the Netherlands; af Department of Cardiothoracic Surgery, Catharina Hospital, Eindhoven, the Netherlands; ag Department of Cardiology, Leiden University Medical Center, Leiden, the Netherlands; ah Department of Cardiothoracic Surgery, Amsterdam University Medical Center location AMC, Amsterdam, the Netherlands; ai Department of Cardiology, University Medical Center Utrecht, Utrecht, the Netherlands; aj Department of Cardiology, HagaZiekenhuis, Den Hague, the Netherlands; ak Department of Cardiology, Isala Klinieken, Zwolle, the Netherlands; al Department of Anesthesia, Amsterdam University Medical Center location AMC, Amsterdam, the Netherlands; am Cardiovascular and Respiratory Physiology, TechMed Center, University of Twente, Enschede, the Netherlands; an Department of Emergency Medicine, Leiden University Medical Center, Leiden, the Netherlands; ao Department of Cardiology, Amsterdam University Medical Center location AMC, Amsterdam, the Netherlands; ap Department of Cardiology, Thorax Center, Erasmus University Medical Center Rotterdam, the Netherlands; aq Department of Methodology & Statistics, Maastricht University, Maastricht, the Netherlands; ar Department of Clinical Epidemiology and Medical Technical Assessment, Maastricht University Medical Center, Maastricht, the Netherlands; a Department of Intensive Care Medicine, Maastricht University Medical Center+, Maastricht University, Maastricht, the Netherlands; b Department of Cardiology, Maastricht University Medical Centre+, Maastricht University, Maastricht, the Netherlands; c Department of Clinical Epidemiology and Medical Technical Assessment, Maastricht University Medical Center+, Maastricht University, Maastricht, the Netherlands; d Department of Cardiology, Isala Clinics, Zwolle, the Netherlands; e Department of Intensive Care, Catharina Hospital, Eindhoven, the Netherlands; f Department of Intensive Care, Leiden University Medical Center, Leiden University, Leiden, the Netherlands; g Department of Intensive Care, Amsterdam University Medical Center, University of Amsterdam, Amsterdam, the Netherlands; h Department of Intensive Care, University Medical Center Utrecht, Utrecht University, Utrecht, the Netherlands; i Department of Intensive Care, St. Antonius Hospital, Nieuwegein, the Netherlands; j Department of Intensive Care, Erasmus Medical Center, Erasmus University, Rotterdam, the Netherlands; k Department of Cardiology, Erasmus Medical Center, Erasmus University, Rotterdam, the Netherlands; l Department of Intensive Care, Maasstad Hospital, Rotterdam, the Netherlands; m Department of Intensive Care, Haga Hospital, The Hague, the Netherlands; n Department of Intensive Care, OLVG, Amsterdam, the Netherlands; o Cardiovascular Research Institute Maastricht (CARIM), Maastricht University, Maastricht, the Netherlands; p Department of Methodology & Statistics, Maastricht University, Maastricht, the Netherlands; q Care and Public Health Research Institute, Maastricht University, Maastricht, the Netherlands; r Department of Cardiothoracic Surgery, Maastricht University Medical Center+, Maastricht University, Maastricht, the Netherlands; s School for Nutrition and Translational Research in Metabolism (NUTRIM), Maastricht University, Maastricht, the Netherlands

**Keywords:** Out-of-hospital cardiac arrest, Extracorporeal cardiopulmonary resuscitation, Informed consent, Ethics

## Abstract

**Background:**

In emergency settings, obtaining timely informed consent is not always feasible, making deferred and waived consent a potential solution. Despite its frequent use in high-risk research, the experiences and opinions of patients and (bereaved) relatives have been scarcely investigated. This study examined their attitudes towards enrolment in the INCEPTION-trial (NCT03101787) on extracorporeal cardiopulmonary resuscitation (ECPR).

**Methods:**

Questionnaires were sent to survivors and (bereaved) relatives who had signed consent forms for follow-up research in the initial INCEPTION-trial. Additionally, relatives where consent was waived were contacted through their general practitioner with a request to participate. Responses included Likert-scale and free-text data, were analysed using descriptive statistics and non-parametric tests.

**Results:**

A total of 32 of 38 (overall response rate 84.2%) sent questionnaires were returned, from 9 survivors, 9 corresponding relatives of these survivors, 6 relatives of non-survivors who provided proxy consent and 8 relatives of non-survivors whose consent was waived. 81.3% of the respondents (strongly) supported alternative consent procedures. No statistically significant differences were found between survivors and non-survivors or ECPR versus conventional cardiopulmonary resuscitation (CCPR). The need for, and challenges of research in an emergency setting were acknowledged. Aftercare contact improved understanding of the trial and helped in bereavement processing.

**Conclusions:**

Overall, patients and (bereaved) relatives had a positive attitude towards waived and deferred consent procedures in high-risk, high-mortality research in the emergency setting. Information provision at a later stage, once the emotional burden has eased, is appreciated.

## Introduction

Informed consent represents the patient's right to be involved in and have authority over the decision-making process concerning their medical care and treatment. Voluntariness and autonomy must be respected, and a deliberate consideration must be undertaken, with the researcher providing sufficient information for the patient to make an informed decision.^1^, ^2^ In emergency research settings, obtaining consent before inclusion may not always be feasible and under certain conditions permission may be granted by the ethical review board to defer consent to a later time.^3^ This may involve either the patient (deferred subject consent) or a representative (deferred proxy consent).^4^ When a patient dies before consent can be obtained, consent is waived according to Dutch law. To avoid selection bias, potentially leading to inaccurate conclusions, it is important to process the data.^4^, ^5^

Despite the legal basis for deferred and waived consent, the principle raises some ethical concerns. Firstly, there is uncertainty about whether representatives can make well-informed decisions. In these challenging situations, relatives are particularly interested in the diagnosis, treatment, and prognosis, and there appears to be little room for explanation regarding participation in a scientific study.^6^ Many representatives base their decisions on the hope for benefit of a new therapy, potentially leading to erroneous consent.^4^, ^6^ Previous research on surrogate decisions indicates error rates around 32%.^7^ Additionally, the retrospective nature of consent may pose an additional psychological burden on patients and their representatives.^4^, ^8^

Previous studies concerning deferred and proxy consent have primarily focused on research with relatively high survival rates and low-risk interventions.^5^, ^9^, ^10^, ^11^, ^12^, ^13^ Research regarding the impact of deferred and waived consent in high-mortality research and involving bereaved individuals, like resuscitation trials, and particularly in treatments as invasive as extracorporeal cardiopulmonary resuscitation (ECPR) is sparse.^14^ To obtain more insight and enhance the deferred and waived consent procedure, we conducted a retrospective questionnaire study on the attitudes of participants and their relatives or bereaved ones enrolled in the INCEPTION-trial. This is a Dutch multicentre trial investigating the use of ECPR versus conventional cardiopulmonary resuscitation (CCPR) in patients with refractory out-of-hospital cardiac arrest (OHCA) and an initial ventricular arrhythmia.^15^

## Methods

The study was performed as ancillary study of the INCEPTION trial (NCT03101787). This multicentre RCT, performed in ten Dutch cardiosurgical centres studied the effectiveness of ECPR for OHCA in comparison with CCPR.^15^ The original study was approved by the Medical Ethical Review Board of Maastricht University (METC 162039) and used deferred consent for data use for all participants. Written informed consent was obtained from a next-of-kin and/or the patient at the earliest convenience, whilst consent was waived in patients who died before consent was obtained.^16^ In the latter cases, next-of-kin were informed about study participation and were given a letter with information about the study. Next-of-kin were invited for a follow-up aftercare conversation, generally within three months after the event. Seven out of the ten study sites participated in this ancillary study.

### Participants

From March 2024 until January 2025, we sent questionnaires to patients and next-of-kin who had consented for participation in further research in the original informed consent form. For deceased patients where consent was waived, their next-of-kin was contacted through the general practitioner of the deceased participant to request their participation before questionnaires were sent out. The questionnaires were adapted to the specific circumstances: patient (patient consent), next-of-kin (proxy consent), next-of-kin of deceased patients (consent waived). Importantly, next-of-kin who provided proxy consent included those whose relative had died before they had the possibility to grant consent. For information, a letter describing the design and results of the INCEPTION trial was included with the questionnaire.

### Ethics

All participants in this ancillary study provided written-informed consent. The protocol for this ancillary study was reviewed by the institutional review board of Maastricht University (METC 2023-0033), which concluded that the psychological burden on individuals completing the questionnaire was not of a significant magnitude to warrant classification under the Medical Research Involving Human Subjects Act (WMO).

### Questionnaires

The formulation of the questions was adjusted for patients, next-of-kin, and bereaved next-of-kin to match their respective situations. First, the respondent’s relationship to the patient, as well their age and gender, were recorded. Subsequently, respondents were asked to respond to six statements related to awareness of study participation, reception of written and/or oral information, understanding of this information, and on their current attitude towards the unsolicited inclusion in the trial, using a 5-point Likert scale (range: strongly disagree – strongly agree); and on three statements related to the timing and amount of this information, using a 3-point Likert scale (too early/too little – just right – too late/too much), with an additional “do not know” option. At the end of the questionnaire, there was free space for additional explanations or comments. The questionnaire was made by our own research team and can be found in Supplements.

### Statistical analysis

Statistical analysis was conducted using IBM SPSS Statistics v28 (SPSS Inc., IL, USA). Quantitative data were analysed using simple descriptive statistics. Likert scale responses were analysed as ordinal data. Groups were compared using the Mann-Whitney *U* test or Kruskal-Wallis test for ordinal data. The proportion of patients answering “do not know” to the statements regarding timing and amount of information was compared between groups using Fisher’s exact test. The significance level was set at *p* < 0.05. No assumptions were made regarding missing data. Due to the small sample size and non-normal distribution, numerical data were summarized as medians with interquartile ranges (IQR). Categorical data were described in percentages.

## Results

### Target cohort

**Fig. 1:**
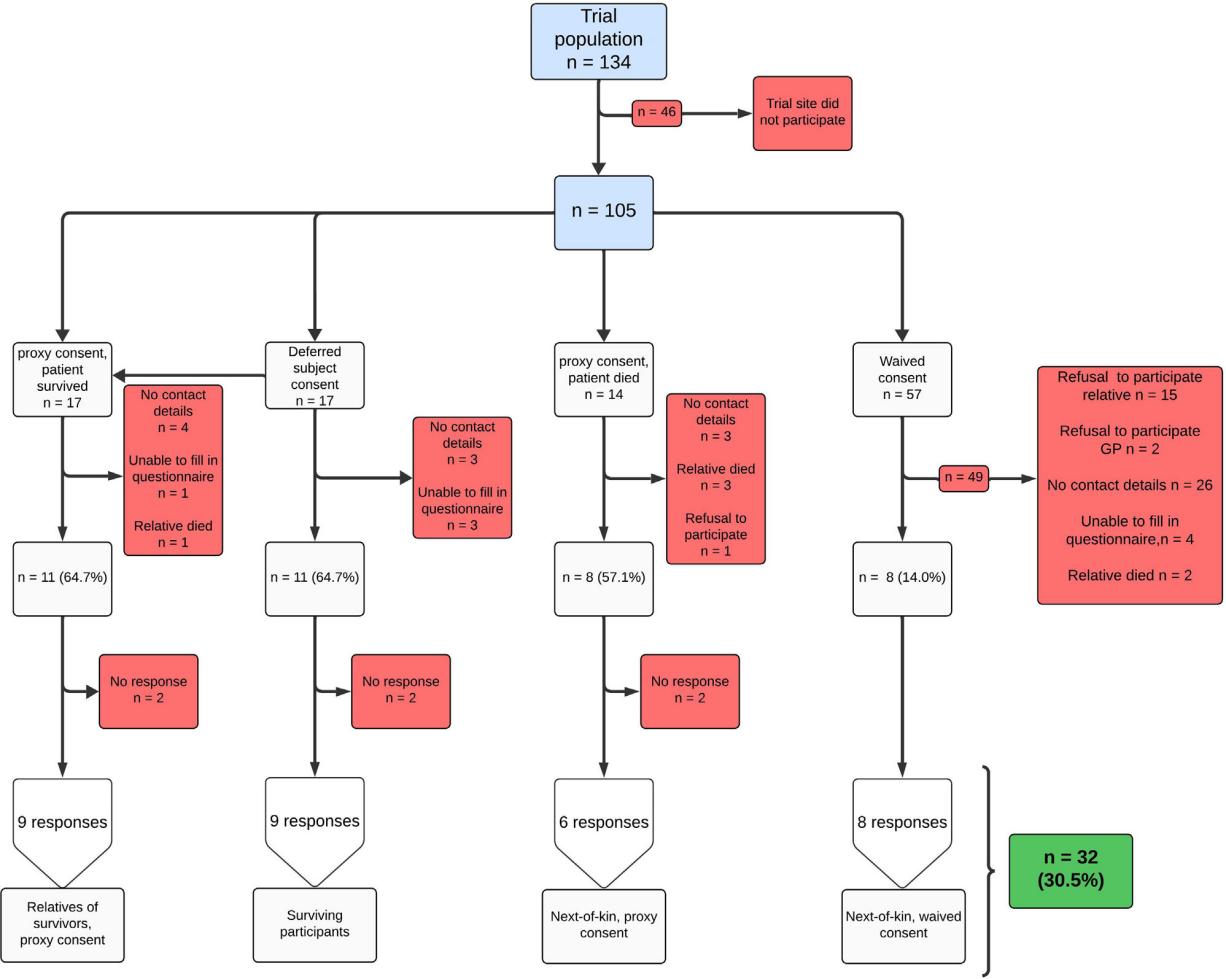
Flowchart of the response rate in the different groupsGP: General practitioner.

The INCEPTION trial included 134 patients across 10 cardiosurgical centres. 7 out of 10 centres were willing to participate in this ancillary study. Eighty-eight patients were enrolled in this trial across these participating centres. In this cohort, overall survival with a favourable neurological outcome after one year was 17/88 (19.3%), 11/49 (22.4%) in patients allocated to ECPR and 6/39 (15.4%) in patients allocated to CCPR. All survivors provided deferred subject consent. Deferred proxy consent was obtained from 31 next-of-kin: 14 were relatives of patients who subsequently died, and 17 were relatives of patients who survived. No representatives withdrew consent. Consent was waived in 57 instances where the patient had died before consent could be obtained. The flow chart and participant categorization are presented in Fig. 1.

### Response rate

In total, we received 32 surveys from 105 potential candidates (30.5%). We were mostly limited by a lack of up-to-date contact information (*n* = 36). Additionally, 16 next-of-kin and 2 general practitioners (GP) declined to participate.

We obtained response from 9 of the 17 surviving patients. For 3 of them, contact information was outdated, 2 did not respond to the questionnaire, and 3 indicated that they were unable to complete the questionnaire due to poor functional status. We obtained responses from 15 out of 31 relatives who provided proxy consent, 7 of them could not be reached due to outdated contact information, 4 relatives were deceased and one declined participation. Four relatives did not respond to our questionnaire. We obtained responses from 9/11 (81.8%) of relatives of surviving patients and from 6/8 (75.0%) of non-surviving patients.

Only 8 out of 57 (14.0%) relatives of participants who were included through waived consent, could be approached, primarily through lack of contact details (*n* = 26). Of the 38 participants or relatives approached, 32 (84.2%) responded. Responses are visualized in Fig. 1.

### Demographics of respondents

**Fig. 2:**
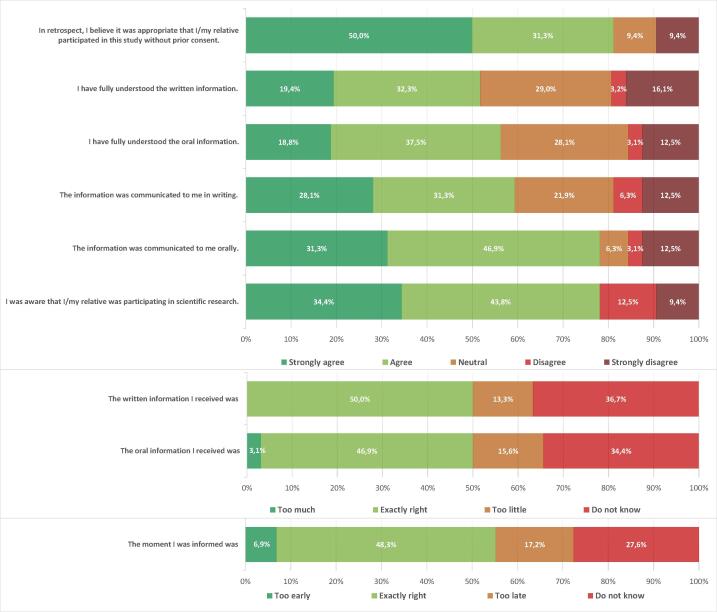
Overview of the opinions on the deferred consent process during the inception trial in all respondents.

The median age of all respondents was 59.0 (54.0; 72.0) years old, and 68.8% were female. The median time between resuscitation and inclusion in this study was 4.6 (4.4; 5.1) years. 3 respondents did not respond to the question about the moment of requesting consent. Two respondents did not answer the question about the quality of the written information, and 1 did not answer the question about the understanding of the written information. All respondents answered all other questions. The distribution of responses to the various questions from all participants is shown in Fig. 2.

### Comprehension of trial participation

Among all respondents 7 (21.9%) disagreed (strongly) that they were aware of trial participation. This number appeared to be the lowest in the patients themselves, and the highest in relatives of patients, where consent was waived, although these differences did not reach statistical significance (*p* = 0.469) (Fig. 3). Three out of 17 responders allocated to ECPR (17.6%) were not aware of trial participation, versus 4 out of 15 (26.7%) allocated to CCPR (Fig. 4). A more detailed breakdown of the responses is available in the Supplementary material, categorized by participant group (Figs. 7–13) and treatment arm (Figs. 14–20).

**Fig. 3:**
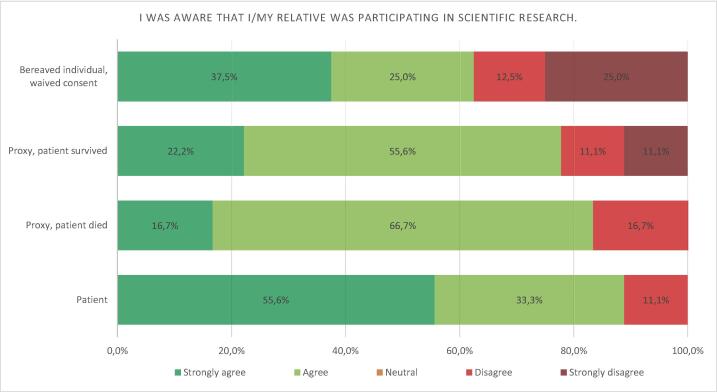
Opinion on awareness of trial participation in the different groups.

**Fig. 4:**
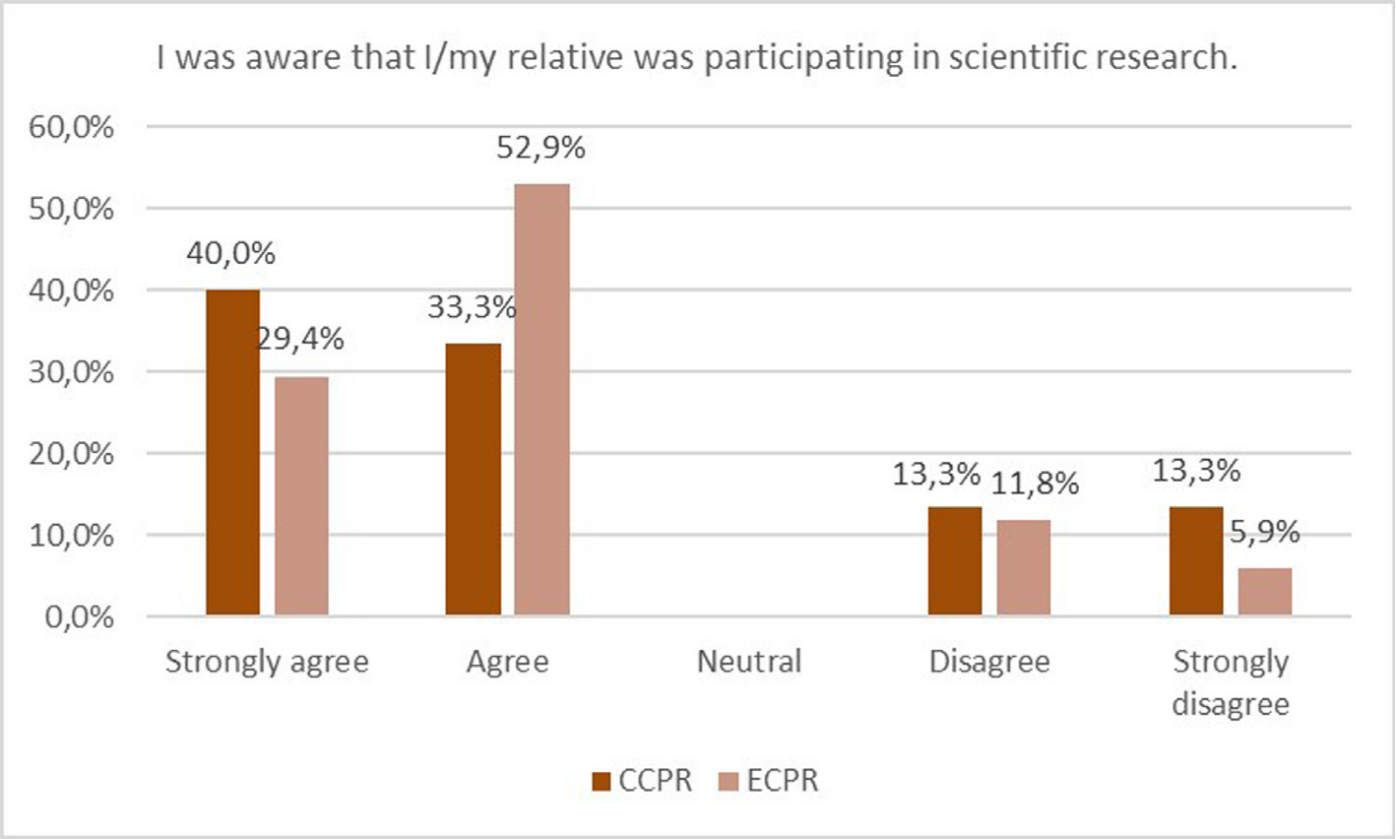
Opinion on awareness of trial participation in the ECPR versus CCPR group.

### Recall and understanding of information and consent

Twenty-five (78.1%) of all respondents indicated they still remember (strongly agree or agree) being informed orally. Eighteen (56.3%) of all respondents had understood the oral information well. Nineteen (59.3%) respondents remembered the provided written information. Sixteen of 31 (51.6%) respondents had understood the written information well. Five (15.6%) out of all respondents mentioned the oral information was too brief, while 4 out of 30 (13.3%) respondents thought the written information was too brief. For 14 out of 29 (48.3%) respondents, the timing was exactly right. Two (6.9%) respondents would have rather been informed later, and 5 (17.2%) would have preferred earlier.

### Inclusion under deferred and waived consent

**Fig. 5:**
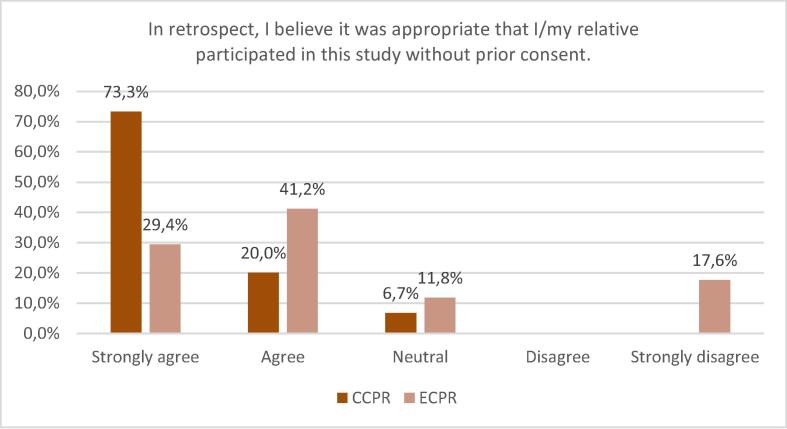
Opinion on alternative consent procedures in the ECPR versus CCPR group.

**Fig. 6:**
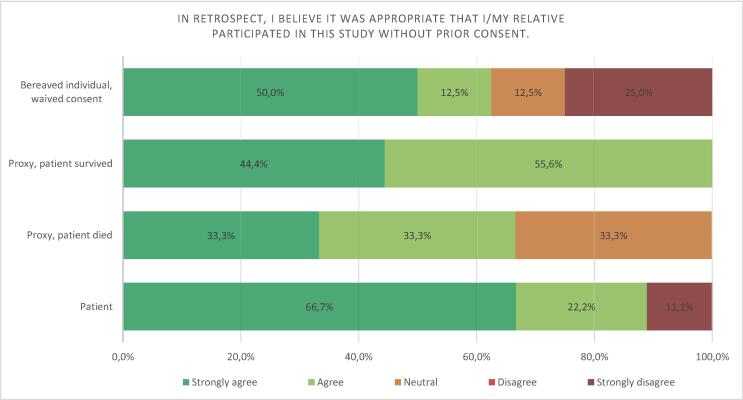
Opinion on alternative consent procedures in the different groups.

Out of 32 respondents 26 (81.3%) strongly agreed or agreed that it was appropriate that their relative was included in the INCEPTION trial using deferred consent. Three (9.4%) respondents, 1 patient and 2 next-of-kin where waived consent was applied, disagreed or strongly disagreed. No statistical difference was found between survivors and non-survivors (*p* = 0.251, Mann-Whitney U) and between the different groups of respondents (*p* = 0.603, Kruskal-Wallis). The distribution of the responses on this question in the ECPR- versus the CCPR-group and in the different groups are illustrated in Fig. 5, Fig. 6 respectively.

### Free text responses

Nineteen respondents further explained their answers in the free text space. Six respondents mentioned the importance of participation for the development of medical care. Three respondents acknowledged the difficulties of consent procedures during emergency care and see it as the best option in these circumstances. Two respondents expressed their disappointment with the study's results and hoped for further research.

In the context of information processing, 5 respondents indicated that emotions hinder retention of information and prioritize other concerns during an ICU-visit. Respondent 14 states: “*They did tell me, but it didn’t really register. My husband had already passed away at that point, and everything just went by in a blur. So I can’t remember exactly, but I know they did everything they could. Thank you*.”

Four respondents expressed their satisfaction with the clarification during follow-up contact. However, 3 respondents indicated a desire for further updates after the last contact. Respondent 9 says: “*Only later did the study and its implications become clear. The final conversation after the death was very enlightening. It is unfortunate that we did not receive further updates on the research.”* Of the 3 respondents who strongly disagreed with the alternative consent procedures, two completed the free-text response. Both indicated that they had no recollection or clear active memory of participating in scientific research. One respondent objected to the prolonged resuscitation time used in this study, citing potential negative consequences for quality of life. An overview of the free text responses is given in Table 2 (Supplements).

## Discussion

This ancillary study found a generally positive attitude (81.3%) among patients, representatives, and bereaved individuals towards deferred and waived consent procedures applied during the INCEPTION trial, which randomized OHCA patients to either ECPR or CCPR. There were only 3 (9.4%) respondents who (strongly) disagreed with this form of consent. Contrary to our hypothesis, bereaved individuals of participants in the INCEPTION trial were not more critical than survivors. Although the sample size was relatively small, these findings suggest an acceptability towards alternative consent procedures in emergency research.

Of note, a significant proportion (21.9%) of our study population could, despite multiple instances of consent or information provision (verbal and written), recall being informed of study participation. Representatives and bereaved individuals indicate that the emotional burden of a resuscitation and an ICU-admission impedes the absorption of information. Most of the participants in the CCPR-group died early at the emergency department. In this group, patient contacts are short and the emotional intensity is high. Since we did not systematically record family conversations at the emergency department, the extent to which relatives were informed about the study is difficult to trace. The brevity of information provision and/or its delivery at a time of heightened emotion could explain the differences in unawareness of trial participation in the CCPR- (26.6%) vs the ECPR-group (17.7%). Importantly, even relatives that were not aware of study participation were in general positive towards the use of deferred consent in the trial. Four respondents indicated their appreciation for the aftercare contact with physicians and researchers. This suggests that providing information through a single letter may be insufficient. In recent years, post-ICU clinics have been increasingly implemented, with positive results for both survivors and their families.^17^, ^18^, ^19^ Despite not routinely provided in the emergency department, there appears to be a demand for aftercare in OHCA patients.

Our results align with earlier studies examining attitudes towards deferred consent in less invasive interventions in resuscitation, or other emergency research. Kamairanen et al.^14^ studied the opinions surrounding deferred consent in patients, their spouses and physicians after their participation or involvement in the consent procedures in a RCT investigating prehospital induction of hypothermia in OHCA. Waived consent was not employed in this study. Furthermore, the researchers also investigated the attitudes towards participation in emergency research in general. All respondents were positive towards their participation in the RCT and taking part in an emergency study without consent if approved by an independent institutional review board.^14^

Other quantitative and qualitative studies have also demonstrated that the majority of patients and proxies accept alternative consent procedures for low-risk interventions, if urgent medical care or the patient’s status impedes the normal informed consent policy.^5^, ^13^, ^9^, ^10^

Reasons for positive attitudes towards deferred and waived consent in our study are consistent with previous research.^9^, ^20^, ^21^, ^22^, ^23^ Most respondents indicated altruistic beliefs, like improving medical care and trust in the treating physicians, as the primary motivation for approval for postponing the consent procedures. Furthermore, the challenges of consent acquisition in emergency circumstances were acknowledged multiple times.

Several factors influence the acceptability of alternative consent procedures. Prior studies showed that the approval of deferring consent was dependent on the risk of the intervention.^20^, ^23^, ^24^, ^25^, ^26^ Patients, representatives and healthcare workers were more critical when studies concern high-risk interventions.^20^, ^23^, ^24^, ^25^, ^26^ Although ECPR is a high-risk intervention, the high acceptability of deferred consent indicated by the respondents in our study may be related to the high mortality risk of standard care.

With the growing popularity of ECPR and increasing public awareness of the potential of ECPR,^27^ attitudes towards potential randomization to the control arm in future ECPR trials may become less favourable. However, since it remains unclear whether the incremental benefit of ECPR outside of highly controlled circumstances reaches a clinically important difference,^28^ equipoise still exists, providing a basis for further research into the real-world effectiveness of ECPR (NCT06805344).

While most, including the current, data suggest that deferred consent is generally well accepted, a similar analysis of the ESCAPE-trial, investigating the effects of thrombectomy in stroke patients, found that a majority of participants disapproved of the application of deferred consent in the ESCAPE-trial or acute stroke trials in general.^11^ However, the latter could not be reproduced in a qualitative survey by van den Bos et al.^12^, where 87% of the interviewed participants found deferred consent acceptable in acute stroke trials. This inconsistency was explained by van den Bos et al. being a consequence of a poor understanding of the randomization process and trial design.^12^ This underlines the importance of evaluating the clarity of the study design after the informed or deferred consent process.

### Strengths and limitations

The participants included in the INCEPTION-trial form a unique study population, given the invasiveness of the treatment and the high mortality risk of the underlying condition. Another strength is the inclusion of respondents from all subgroups providing a comprehensive overview of the previously underexplored attitudes towards deferred consent in OHCA studies. In particular, this paper offers the unique perspective of relatives of participants in which consent was waived. In addition, we conducted both a quantitative and qualitative assessment of the outcome measures, which gave us a broader insight into their responses.

Notwithstanding, this study has several limitations. Firstly, the questionnaire was developed by our research group without external validation or review amongst stakeholders. In addition, one of the research challenges was to minimize emotional burden. Consequently, the investigators timed the study to take place a significant period after the resuscitation. The long time between study inclusion and the questionnaire inevitably introduced recollection bias, which presumably explains the relative high percentage of respondents with no recall of the recall of the quality of the given information. The legal requirement to involve the general practitioner in approaching subjects who had not yet consented to follow-up research limited the number of relatives where consent was waived may have introduced selection bias.

Finally, the sample size for this study was small and the overall response rate (32 out of 105 potential surveys), is low. Nevertheless, the response rate of the individuals approached was high (84.2%) and the responses were broadly homogenous, increasing confidence in the conclusions of our research.

## Conclusions

This study demonstrates a general approval for waived and deferred consent procedures in the INCEPTION trial, which confirms the acceptability of using these approaches in high-risk, high-mortality research for patients, their relatives/representatives, and bereaved individuals of deceased participants. The guidelines for alternative consent procedures of the Helsinki principles and the Dutch Central Committee on Research Involving Human Subjects appear to be an acceptable framework for research in (emergency) settings where a regular informed consent process is not feasible. In such a context, additional information provision at a later stage, when emotional burdens are reduced, is valued and could potentially be implemented as standard practice.

## Declaration of AI and AI-assisted technologies in the writing process

No Artificial Intelligence assisted tools were used.

## CRediT authorship contribution statement

**Stijn E.D.M. Eussen:** Writing – original draft, Methodology, Investigation, Formal analysis. **Anina F. van de Koolwijk:** Writing – review & editing, Methodology, Investigation, Formal analysis. **Thijs S.R. Delnoij:** Writing – review & editing, Validation, Data curation, Conceptualization. **Martje M. Suverein:** Writing – review & editing, Validation, Investigation, Data curation, Conceptualization. **Brigitte A.B. Essers:** Writing – review & editing, Validation, Methodology. **Renicus C. Hermanides:** Validation, Resources, Investigation. **Luuk C. Otterspoor:** Validation, Resources, Investigation. **Carlos V. Elzo Kraemer:** Validation, Resources, Investigation. **Alexander P.J. Vlaar:** Validation, Resources, Investigation. **Joris J. van der Heijden:** Validation, Resources, Investigation, Conceptualization. **Erik Scholten:** Validation, Resources, Investigation. **Corstiaan A. den Uil:** Validation, Resources, Investigation. **Dinis Dos Reis Mirada:** Validation, Resources. **Sakir Akin:** Validation, Resources, Investigation. **Jesse de Metz:** Validation, Resources, Investigation. **Iwan van der Horst:** Writing – review & editing, Validation, Supervision, Methodology. **Bjorn Winkens:** Validation, Methodology, Formal analysis. **Jos G. Maessen:** Writing – review & editing, Validation, Supervision, Resources, Methodology, Conceptualization. **Roberto Lorusso:** Writing – review & editing, Validation, Supervision, Resources, Methodology, Conceptualization. **Marcel C.G. van de Poll:** Writing – review & editing, Writing – original draft, Validation, Methodology, Investigation, Funding acquisition, Formal analysis, Conceptualization. **George J. Brandon Bravo:** **Luuk Otterspoor:** Validation, Resources, Investigation. **Corstiaan den Uil:** Validation, Resources, Investigation. **Tim Jansen:** **Bas van den Bogaard:** **Marijn Kuijpers:** **Ka Yan Lam:** **José M. Montero Cabezas:** **Antoine H.G. Driessen:** **Saskia Z.H. Rittersma:** **Bram G. Heijnen:** **Dinis Dos Reis Miranda:** Validation, Resources. **Gabe Bleeker:** **Renicus S. Hermanides:** Validation, Resources, Investigation. **Jorge Lopez Matta:** **Susanne Eberl:** **Dirk W. Donker:** **Robert J. van Thiel:** **Oene van Meer:** **José Henriques:** **Karen C. Bokhoven:** **Henrik Endeman:** **Jeroen J.H. Bunge:** **Martine E. Bol:** **Brigitte Essers:** Writing – review & editing, Validation, Methodology. **Patrick W. Weerwind:** .

## Funding

This research did not receive any specific grant from funding agencies in the public, commercial, or not-for-profit sectors. The original INCEPTION-trial was funded by the Netherlands Organization for Health Research and Development (ID: 843001707) and Maquet Cardiopulmonary [Getinge].

## Declaration of competing interest

Roberto Lorusso reports consulting fees from Abiomed, and participates in an advisory board of Xenios, not related to this work. All other authors have no conflicts of interest to declare.
