# Supplementary material for: Attitudes of patients and family members towards deferred and waived consent in ECPR research, an ancillary study of the INCEPTION trial
Source: Resusc Plus. 2026 Jan 21;28:101239. doi: 10.1016/j.resplu.2026.101239 (PMC12906210; doi:10.1016/j.resplu.2026.101239)
Supplement: Supplementary Data 1 [file mmc1.pdf]

Supplementary material

Table of contents

Supplement 1 – Overview of the opinion on the other questions in the different group of responders 2

Supplement 2 - *Overview of the opinions on the other questions in the ECPR group vs the CCPR group* 6

Supplement 3 – Baseline characteristics of the respondents 10

Supplement 4 – Overview of the free text responses 11

Supplement 5 – Word cloud of the free text responses 12

Supplement 6 – Information letter 13

Supplement 7 – Questionnaire 15

Appendix A – INCEPTION investigators 18

Supplement 1 – Overview of the opinion on the other questions in the different group of responders

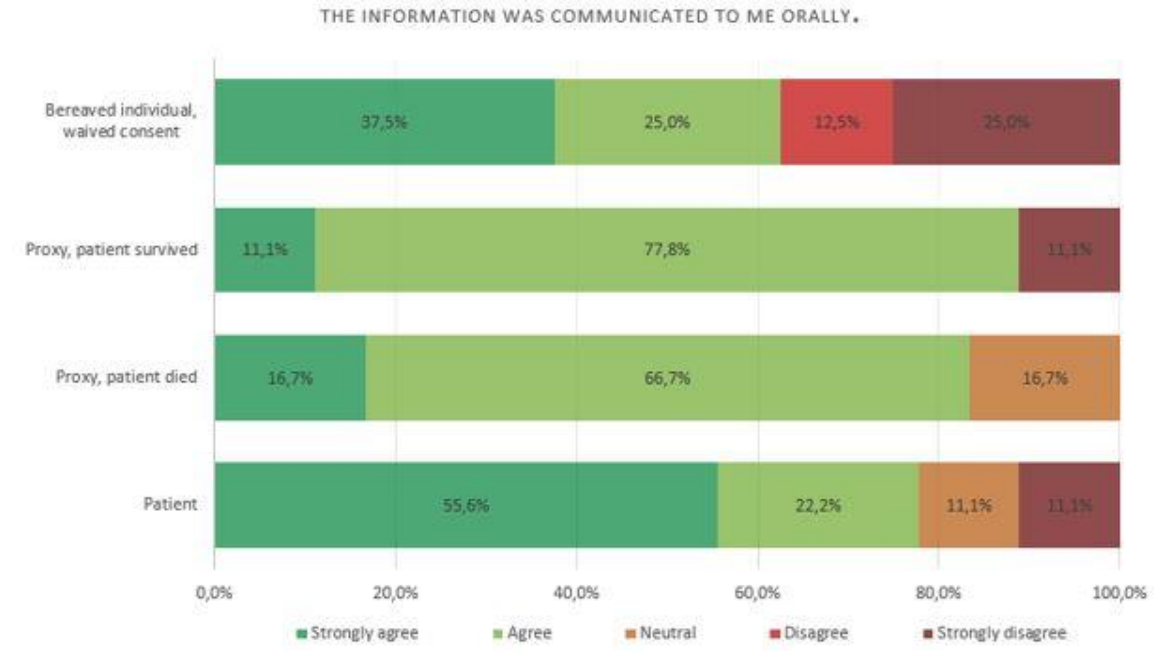

Figure 7s, overview of the opinions of on whether oral communication occurred in the different groups

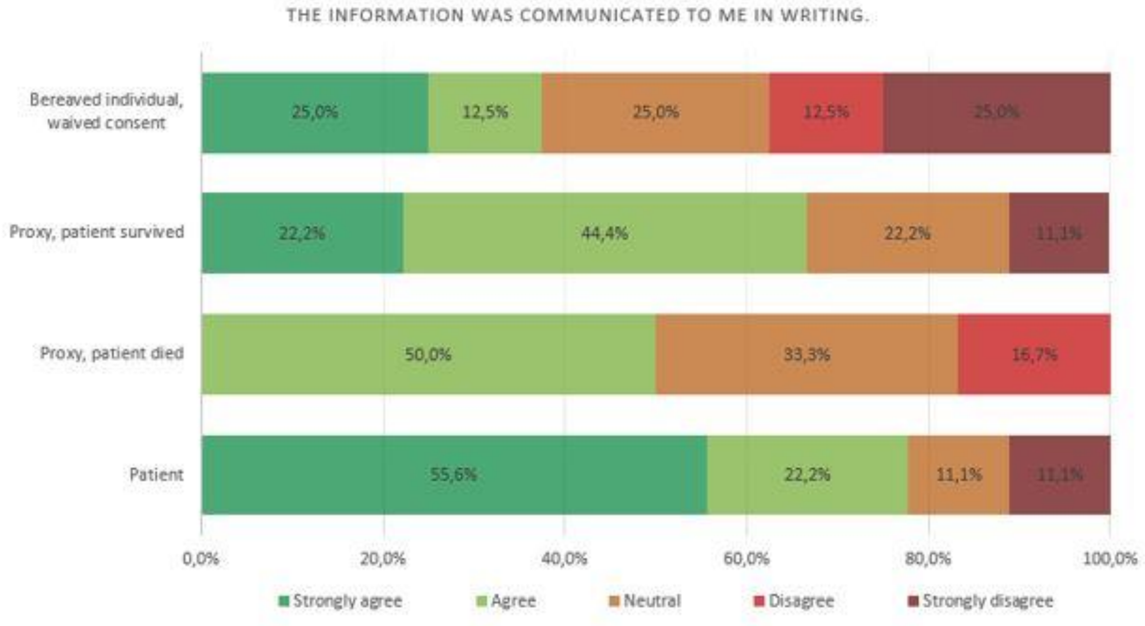

Figure 8s, overview of the opinions on whether written communication occurred in the different groups

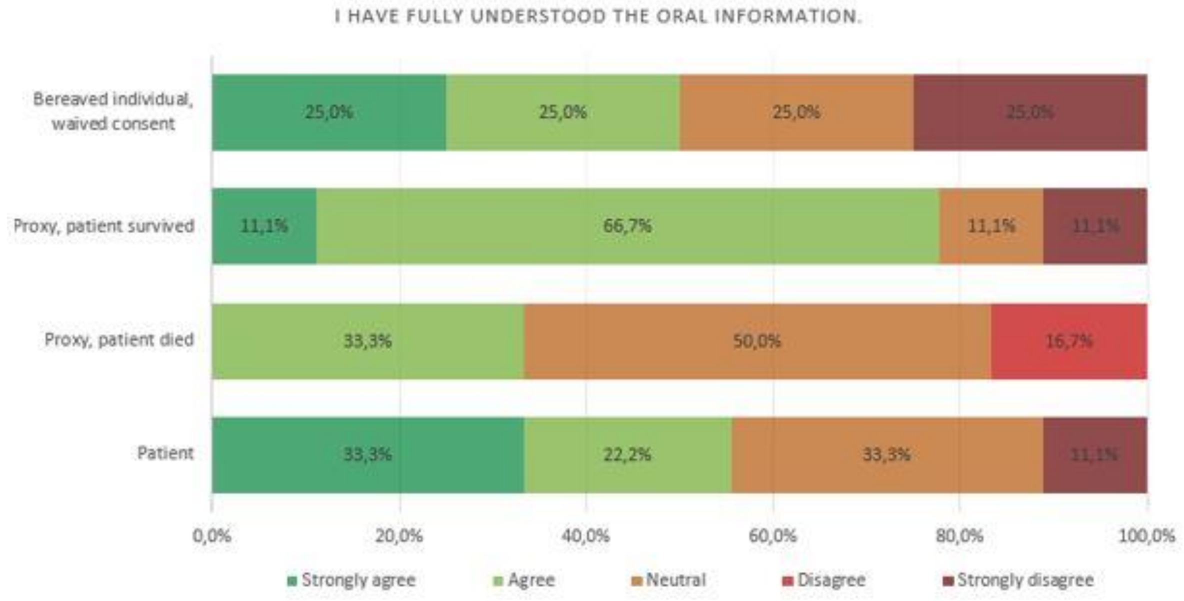

Figure 9s, overview of the opinions on the comprehension of oral communication in the different groups

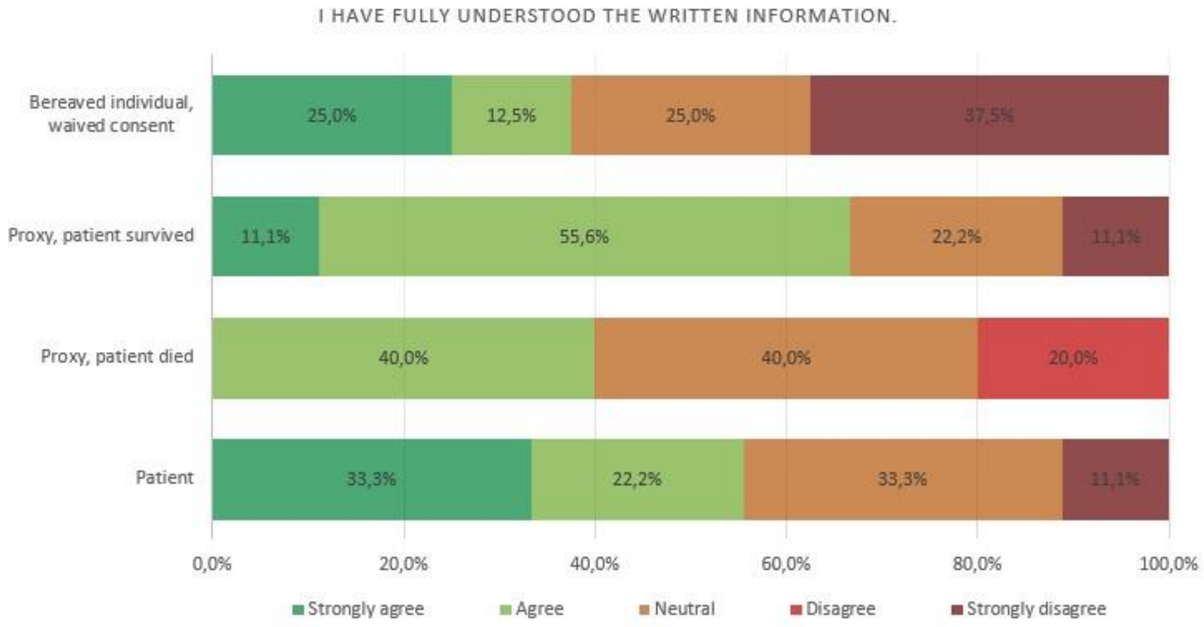

Figure 10s, overview of the opinions on the comprehension of the written communication in the different groups

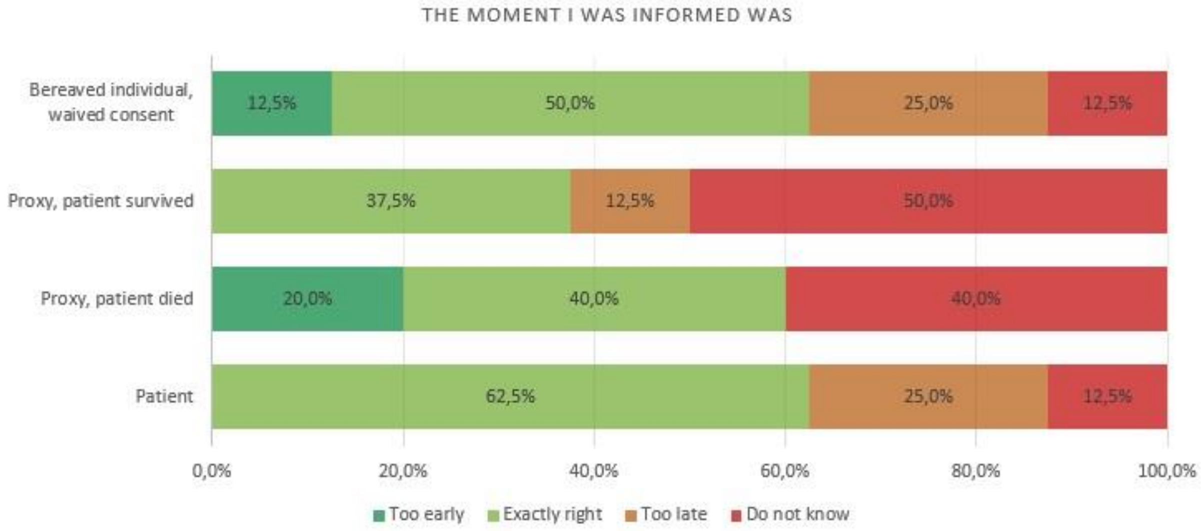

Figure 11s, overview of the opinions on the moment of information in the different groups

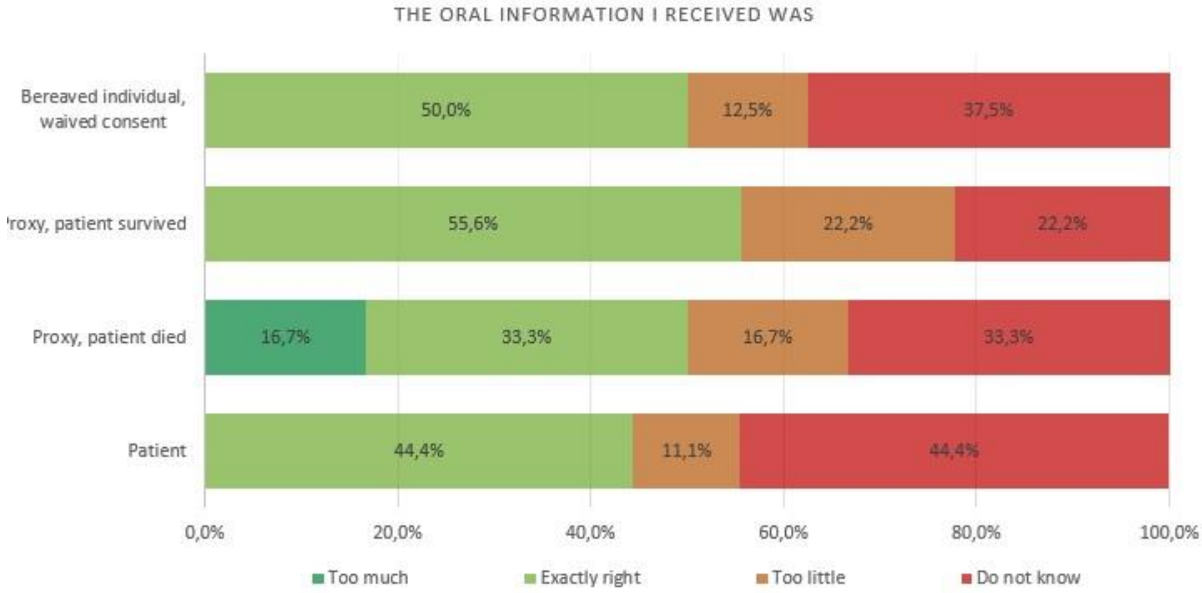

Figure 12s, overview of the opinions on the quality of the oral information in the different groups

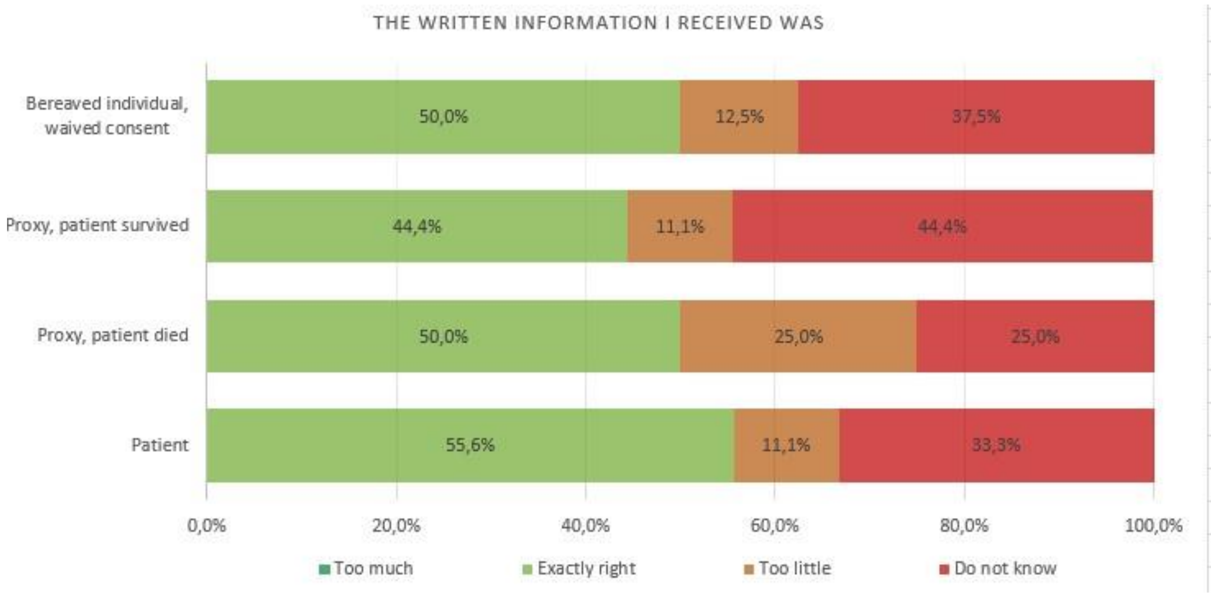

Figure 13s, overview of the opinions on the quality of the written information in the different groups

Supplement 2 - *Overview of the opinions on the other questions in the ECPR group vs the CCPR group*

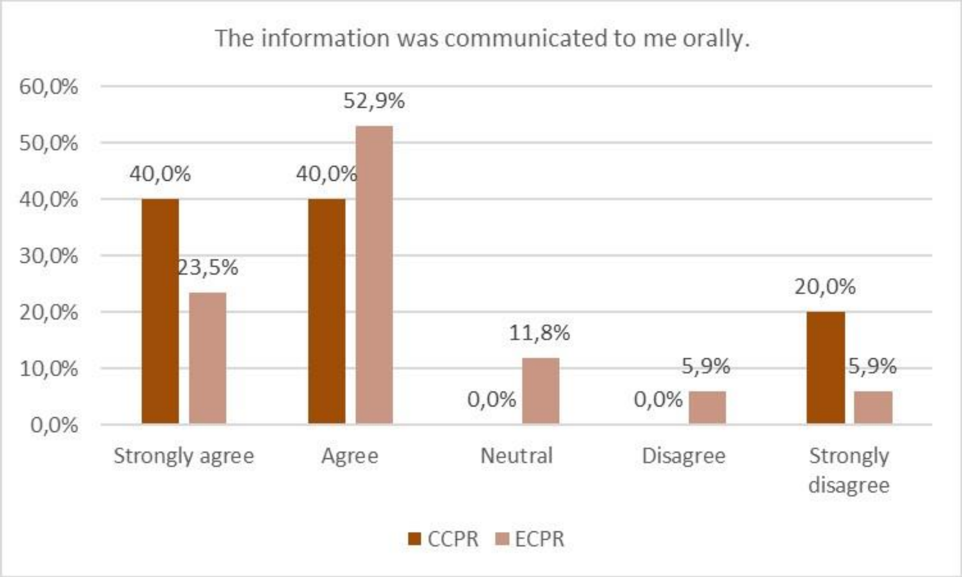

Figure 14s, overview of the opinions on whether oral communication occurred in the ECPR group versus the CCPR group

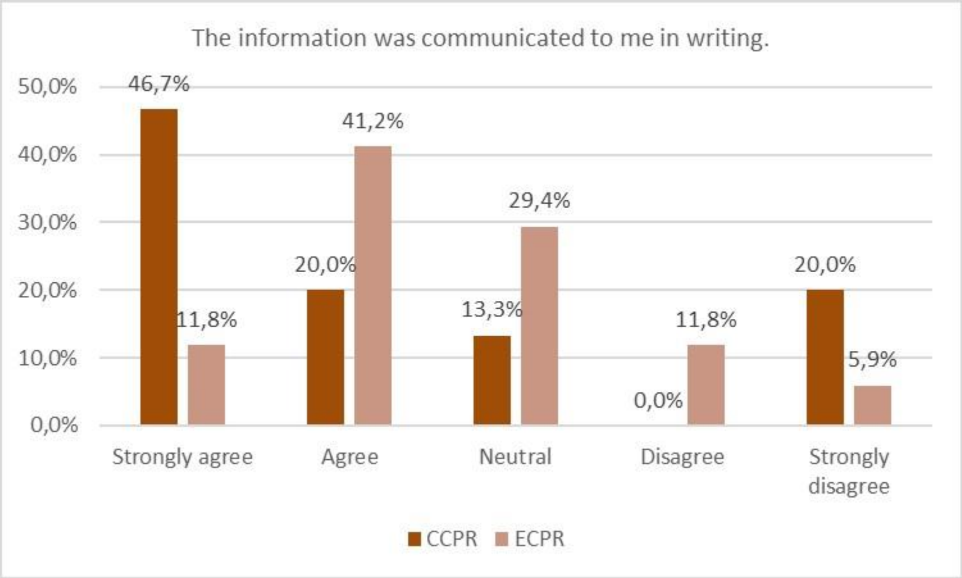

Figure 15s, overview of the opinions on whether written communication occurred in the ECPR group versus the CCPR group

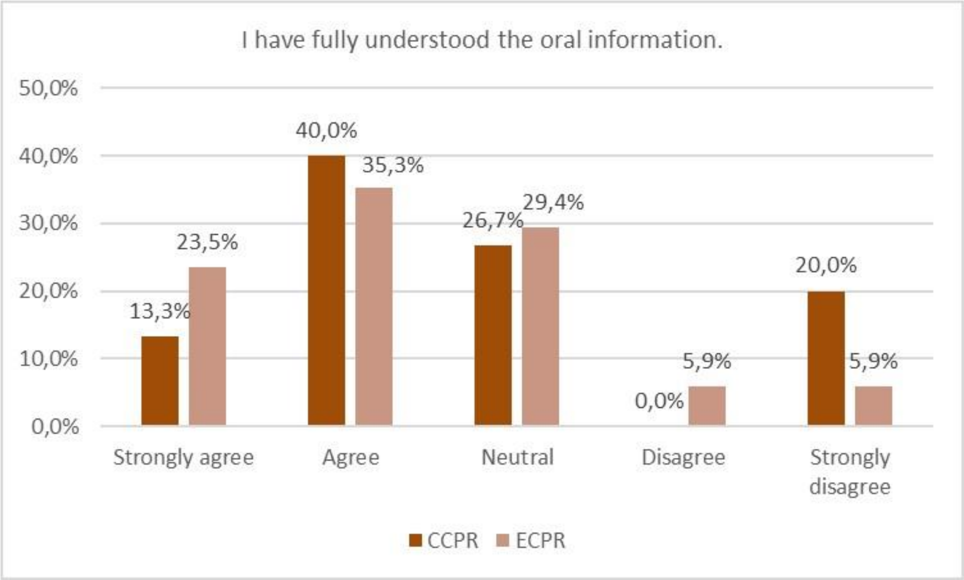

Figure 16s, overview of the opinions on the comprehension of oral information in the ECPR group versus the CCPR group

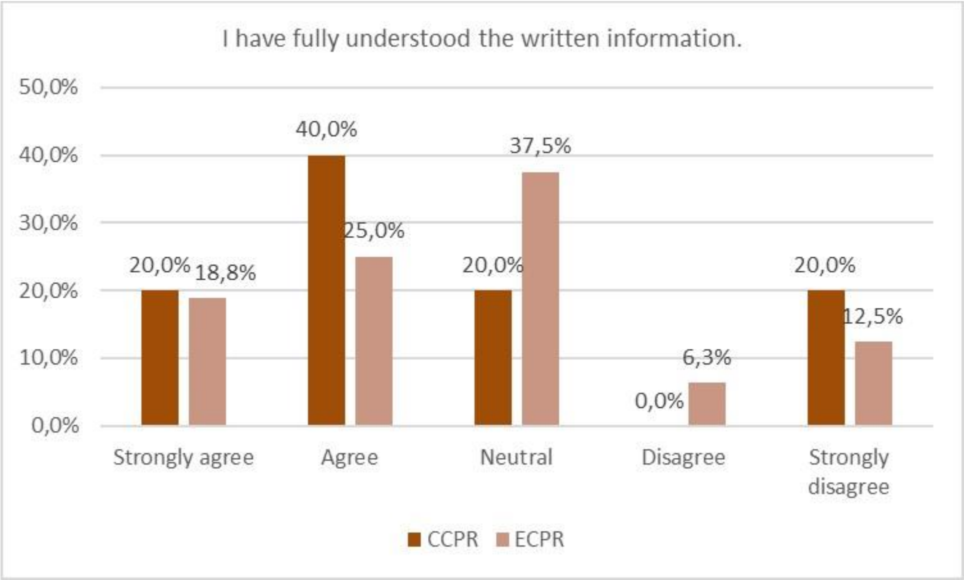

Figure 17s, overview of the opinions on the comprehension of written information in the ECPR group versus the CCPR group

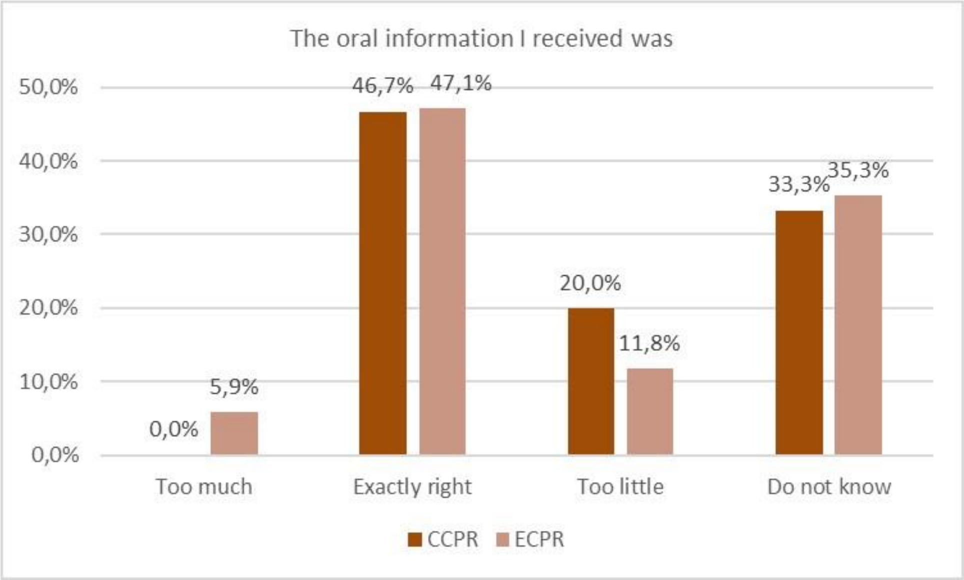

Figure 18s, overview of the quality of the oral information in the ECPR group versus the CCPR group

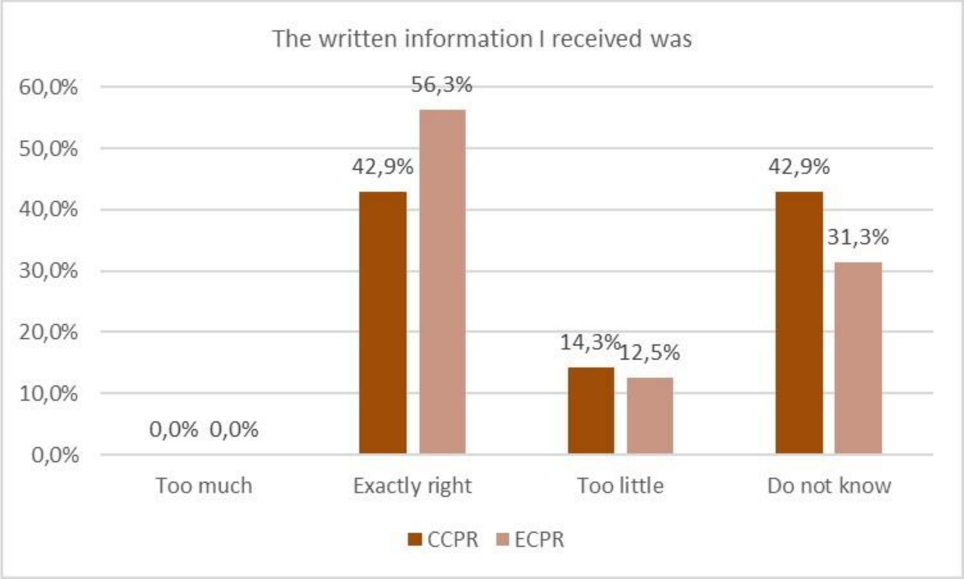

Figure 19s, overview of the opinions on the quality of the written information in the ECPR group versus the CCPR group

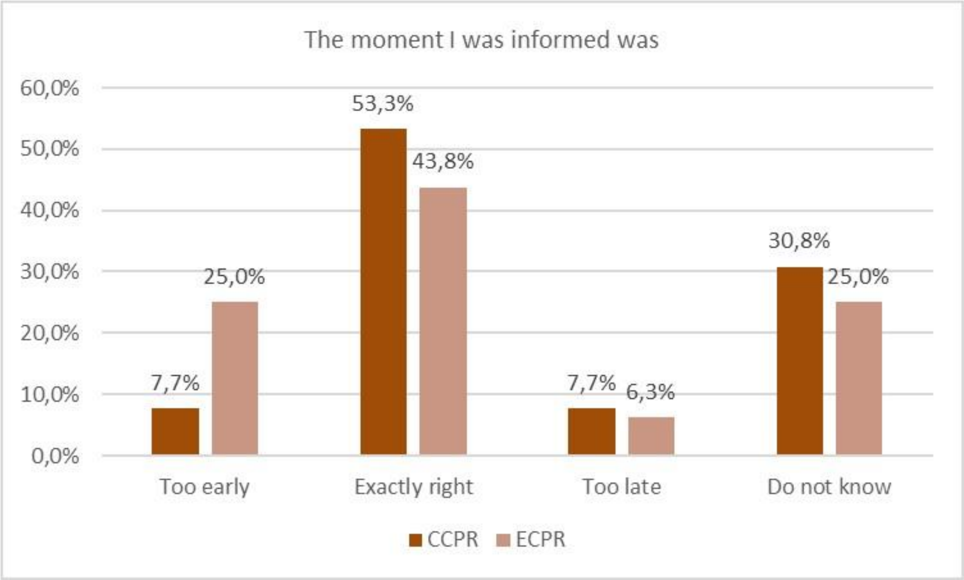

Figure 20s, overview of the opinions on the moment of information in the ECPR group versus the CCPR group

Supplement 3 – Baseline characteristics of the respondents

	Patient (N=9)	Proxy survived (N=9)	Proxy deceased (N=6)	Waived (N=8)	Total (N=32)
Time since inclusion (years)	4.7	5.0	4.5	4.4	4.6
Relation to patient	n/a				
Spouse		8 (88.9)	5 (83.3)	7 (87.5)	20 (87.0)
Child		1 (11.1)	1 (16.7)	1 (12.5)	3 (13.0)
Gender (%)					
Male	88.9	11.1	0.0	12.5	31.3
Female	11.1	88.9	100.0	87.5	68.8
					(p< 0.001)
Age at time of inclusion (years)	63.0	59.0	58.0	60.5	59.0
Treatment (%)	44.4	33.3	83.3	62.5	53.1
ECPR	55.6	66.7	16.7	37.5	46.9
CCPR					(p= 0.244)

Table 1s, baseline characteristics of the respondents

Supplement 4 – Overview of the free text responses

Category	Number of respondents	Details
Total	19	
Altruism	6	Mentioned the importance for the development of medical care
Consent procedures in emergency setting	3	Acknowledged challenges of informed consent procedures in the emergency setting
Study results	2	Expressed disappointment with the results in the intervention group
Information processing/ no active memory	5	Indicated emotions hinder retention of information and prioritize other concerns
Follow-up contact	4	Expressed satisfaction with follow-up contact
Updates	3	Mentioned a desire for further updates after the last contact
Quality of life	1	Objected the prolonged resuscitation time and potential negative implications on quality of life

Table 2s, overview of the free text responses

Supplement 5 – Word cloud of the free text responses

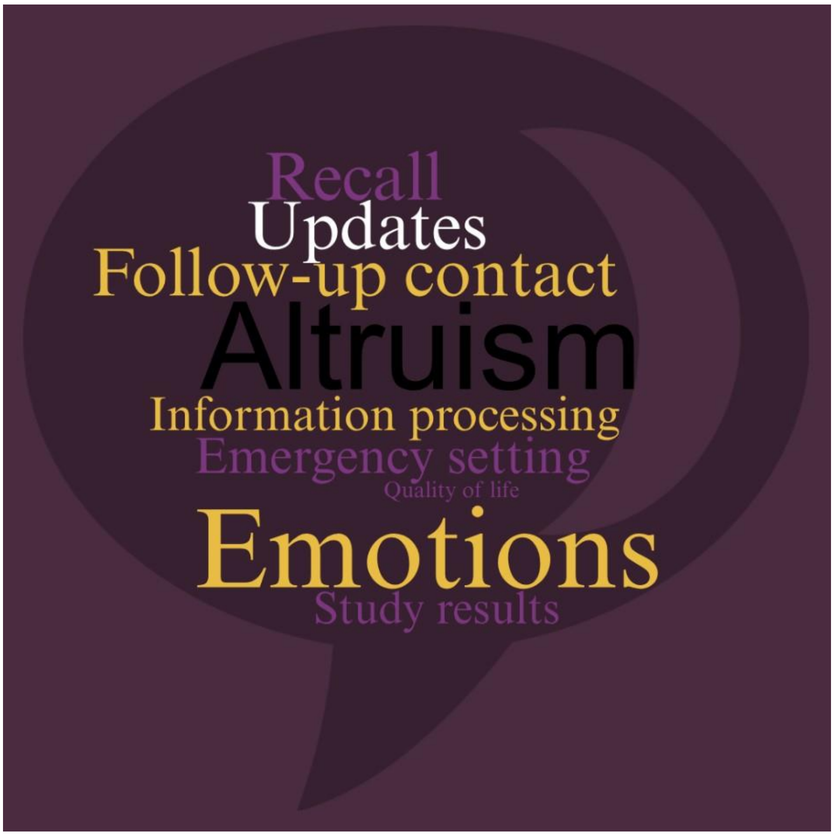

Figure 21s, word cloud of the free text responses

Maastricht UMC+

Supplement 6 – Information letter

Dear Sir/Madam,

On <admission date>, your relative <Mr/Ms> <name> was treated at <hospital> following a sudden cardiac arrest.

You may recall that at the time you were informed that a scientific study was being conducted to examine whether the use of a heart-lung machine (extracorporeal circulation) during resuscitation would improve survival rates. To investigate this, patients were randomly assigned to either receive or not receive treatment with a heart-lung machine during resuscitation. Ten hospitals across the country participated in this study.

Because in emergency situations there is no time to request prior consent for participation in scientific research, the Medical Ethics Review Committee (METC) granted approval to include patients without prior consent. This was possible because, at the time of the study, it was entirely uncertain which of the two existing treatments would lead to better outcomes.

Your relative also participated in this study and was resuscitated <with/without> a heart-lung machine. You were informed orally about this by the attending physician or a researcher and gave consent on behalf of your relative for participation. Later, your relative also provided written consent for participation in the study.

In total, 134 patients participated in the study, of whom 55 were treated with a heart-lung machine. After six months, 24 of the 134 patients (18%) were still alive. The survival rates were the same for patients resuscitated with and without the heart-lung machine. The randomization therefore had no beneficial or adverse effect on your relative's chances of survival. The conclusion of the study is that it is not useful to employ a heart-lung machine as a standard intervention for patients suffering cardiac arrest outside the hospital. However, further research is required to determine whether in certain patients or specific situations the heart-lung machine may still provide added value.

Because we consider it important to inform participants in scientific research and their relatives as transparently as possible, we wish to share the outcomes of this study with you.

Furthermore, we would like to know whether the information provided to you during what was undoubtedly a very difficult time was sufficiently clear, and how you reflect on the fact that your relative participated in this scientific study in this way. For this purpose, we kindly ask you to complete a questionnaire.

If you are willing to complete this questionnaire, your experiences will help us formulate recommendations for other researchers who may in the future conduct studies in emergency situations where it is not possible to obtain consent in advance.

If you have any questions about this study, or if you would like more information before deciding whether or not to participate, please contact one of the researchers.

The results of the questionnaires will be processed anonymously. Apart from the principal

Maastricht UMC+

investigator at Maastricht UMC+, no one will be able to trace the responses back to you personally.

We sincerely thank you and hope you are willing to complete the questionnaire.

Yours faithfully,
on behalf of the entire research team,

Drs. S.E.D.M. Eussen
Drs. A.F. van de Koolwijk
Dr. M.C.G. van de Poll
Department of Intensive Care, Maastricht UMC +

Maastricht UMC+

Supplement 7 – Questionnaire

I have read the information letter and I consent to the use of my questionnaire responses for the stated purpose:

[ ] Yes [ ] No

What is your relationship to the patient?

[ ] Partner [ ] Child [ ] Other, namely: ____________

What is your gender?

[ ] Male [ ] Female [ ] Other

What is your age? __________

1. I am aware that my relative participated in a scientific study:

[ ] Strongly agree [ ] Agree [ ] Neutral [ ] Disagree [ ] Strongly disagree

2. This information was communicated to me orally:

[ ] Strongly agree [ ] Agree [ ] Neutral [ ] Disagree [ ] Strongly disagree

3. This information was communicated to me in writing:

[ ] Strongly agree [ ] Agree [ ] Neutral [ ] Disagree [ ] Strongly disagree

4. I understood the oral information provided to me at the time:

[ ] Strongly agree [ ] Agree [ ] Neutral [ ] Disagree [ ] Strongly disagree

5. I understood the written information provided to me at the time:

[ ] Strongly agree [ ] Agree [ ] Neutral [ ] Disagree [ ] Strongly disagree

6. The oral information I received was:

[ ] Insufficient [ ] Adequate [ ] Too much [ ] Don't know

7. The written information I received was:

[ ] Insufficient [ ] Adequate [ ] Too much [ ] Don't know

8. In retrospect, I believe it was appropriate that my relative participated in this study without prior consent:

[ ] Strongly agree [ ] Agree [ ] Neutral [ ] Disagree [ ] Strongly disagree

9. I would have preferred to be informed about the study at a different time:

[ ] Earlier [ ] At the right time [ ] Later [ ] Don't know

10. I have no objection to being contacted by telephone or in writing for follow-up questions about this study:

[ ] No objection [ ] Objection

11. If you would like to elaborate on any of your answers above, or if you wish to share anything else with us, please use the space below:

____

____

____

____

____

____

____

____

____

Appendix A – INCEPTION investigators

Martje M. Suverein, M.D.	Department of Intensive Care, Maastricht University Medical Center, Maastricht, the Netherlands
Thijs S.R. Delnoij, M.D.	Department of Intensive Care, Maastricht University Medical Center, Maastricht, the Netherlands
Roberto Lorusso, M.D., Ph.D.	Department of Cardiothoracic Surgery, Maastricht University Medical Center, Maastricht, the Netherlands
George J. Brandon Bravo Bruinsma, M.D., Ph.D.	Department of Cardiothoracic Surgery, Isala Klinieken, Zwolle, the Netherlands
Luuk Otterspoor, M.D., Ph.D.	Department of Intensive Care, Catharina Hospital, Eindhoven, the Netherlands
Carlos V. Elzo Kraemer, M.D., Ph.D.	Department of Intensive Care, Leiden University Medical Center, Leiden, The Netherlands
Alexander P.J. Vlaar, M.D., Ph.D.	Department of Intensive Care, Amsterdam University Medical Center location AMC, Amsterdam, the Netherlands
Joris J. van der Heijden, M.D.	Department of Intensive Care, University Medical Center Utrecht, Utrecht, The Netherlands
Erik Scholten, M.D.	Department of Intensive Care, St. Antonius Hospital, Nieuwegein, The Netherlands
Corstiaan den Uil, M.D., Ph.D.	Department of Intensive Care, Erasmus Medical Center, Rotterdam, The Netherlands
Tim Jansen, M.D., Ph.D.	Department of Intensive Care, HagaZiekenhuis, Den Hague, The Netherlands
Bas van den Bogaard, M.D., Ph.D.	Department of Intensive Care, OLVG, Amsterdam, The Netherlands
Marijn Kuijpers, M.D.	Department of Intensive Care, Isala Klinieken, Zwolle, the Netherlands
Ka Yan Lam, M.D.	Department of Cardiothoracic Surgery, Catharina Hospital, Eindhoven, the Netherlands
José M. Montero Cabezas, M.D.	Department of Cardiology, Leiden University Medical Center, Leiden, The Netherlands
Antoine H.G. Driessen, M.D., Ph.D.	Department of Cardiothoracic Surgery, Amsterdam University Medical Center location AMC, Amsterdam, The Netherlands
Saskia Z.H. Rittersma, M.D., Ph.D.	Department of Cardiology, University Medical Center Utrecht, Utrecht, The Netherlands

Bram G. Heijnen, M.D.	Department of Intensive Care, St. Antonius Hospital, Nieuwegein, The Netherlands
Dinis Dos Reis Miranda, M.D., Ph.D.	Department of Intensive Care, Erasmus Medical Center, Rotterdam, The Netherlands
Gabe Bleeker, M.D., Ph.D.	Department of Cardiology, HagaZiekenhuis, Den Hague, The Netherlands
Jesse de Metz, M.D., Ph.D.	Department of Intensive Care, OLVG, Amsterdam, The Netherlands
Renicus S. Hermanides, M.D., Ph.D.	Department of Cardiology, Isala Klinieken, Zwolle, the Netherlands
Jorge Lopez Matta, M.D.	Department of Intensive Care, Leiden University Medical Center, Leiden, The Netherlands
Susanne Eberl, M.D., Ph.D.	Department of Anesthesia, Amsterdam University Medical Center location AMC, Amsterdam, The Netherlands
Dirk W. Donker, M.D., Ph.D.	Cardiovascular and Respiratory Physiology, TechMed Center, University of Twente, Enschede, The Netherlands<br>Department of Intensive Care, University Medical Center Utrecht, Utrecht, The Netherlands
Robert J. van Thiel, M.D.	Department of Intensive Care, Erasmus Medical Center, Rotterdam, The Netherlands
Sakir Akin, M.D., Ph.D.	Department of Intensive Care, HagaZiekenhuis, Den Hague, The Netherlands
Oene van Meer, M.D.	Department of Emergency Medicine, Leiden University Medical Center, Leiden, The Netherlands
José Henriques, M.D., Ph.D.	Department of Cardiology, Amsterdam University Medical Center location AMC, Amsterdam, The Netherlands
Karen C. Bokhoven, M.D.	Department of Intensive Care, Erasmus Medical Center, Rotterdam, The Netherlands
Henrik Endeman, M.D., Ph.D.	Department of Intensive Care, Erasmus Medical Center, Rotterdam, The Netherlands
Jeroen J.H. Bunge, M.D.	Department of Intensive Care, Erasmus Medical Center, Rotterdam, The Netherlands<br>Department of cardiology, Thorax Center, Erasmus University Medical Center Rotterdam, The Netherlands
Martine E. Bol,	Department of Intensive Care, Maastricht University Medical Center, Maastricht, the Netherlands

Bjorn Winkens, Ph.D.	Department of Methodology & Statistics, Maastricht University, Maastricht, The Netherlands
Brigitte Essers, Ph.D.	Department of Clinical Epidemiology and Medical Technical Assessment, Maastricht University Medical Center, Maastricht, the Netherlands
Patrick W. Weerwind, CCP, Ph.D.	Department of Cardiothoracic Surgery, Maastricht University Medical Center, Maastricht, the Netherlands
Jos G. Maessen, M.D., Ph.D.	Department of Cardiothoracic Surgery, Maastricht University Medical Center, Maastricht, the Netherlands
Marcel C.G. van de Poll, M.D., Ph.D.	Department of Intensive Care, Maastricht University Medical Center, Maastricht, the Netherlands
